# A dipeptide transporter from the arbuscular mycorrhizal fungus *Rhizophagus irregularis* is upregulated in the intraradical phase

**DOI:** 10.3389/fpls.2014.00436

**Published:** 2014-09-03

**Authors:** Simone Belmondo, Valentina Fiorilli, Jacob Pérez-Tienda, Nuria Ferrol, Roland Marmeisse, Luisa Lanfranco

**Affiliations:** ^1^Department of Life Sciences and Systems Biology, University of TorinoTorino, Italy; ^2^Istituto per la Protezione Sostenibile delle Piante, Consiglio Nazionale delle RicercheTorino, Italy; ^3^Departamento de Microbiología del Suelo y Sistemas Simbióticos, Consejo Superior de Investigaciones CientificasGranada, Spain; ^4^Ecologie Microbienne, UMR CNRS 5557 - USC INRA 1364, Université Lyon 1, Université de LyonVilleurbanne, France

**Keywords:** arbuscular mycorrhizal fungi, organic nitrogen, dipeptide transporter, *Rhizophagus irregularis*, symbiosis

## Abstract

Arbuscular mycorrhizal fungi (AMF), which form an ancient and widespread mutualistic symbiosis with plants, are a crucial but still enigmatic component of the plant micro biome. Nutrient exchange has probably been at the heart of the success of this plant-fungus interaction since the earliest days of plants on land. To characterize genes from the fungal partner involved in nutrient exchange, and presumably important for the functioning of the AM symbiosis, genome-wide transcriptomic data obtained from the AMF *Rhizophagus irregularis* were exploited. A gene sequence, showing amino acid sequence and transmembrane domains profile similar to members of the PTR2 family of fungal oligopeptide transporters, was identified and called *RiPTR2*. The functional properties of *RiPTR2* were investigated by means of heterologous expression in *Saccharomyces cerevisiae* mutants defective in either one or both of its di/tripeptide transporter genes *PTR2* and *DAL5*. These assays showed that RiPTR2 can transport dipeptides such as Ala-Leu, Ala-Tyr or Tyr-Ala. From the gene expression analyses it seems that *RiPTR2* responds to different environmental clues when the fungus grows inside the root and in the extraradical phase.

## Introduction

Nitrogen (N) is quantitatively the most important soil nutrient for plant growth and productivity. Plants acquire N not only as nitrate and ammonium that are converted to amino acids in the root or shoot tissues, but also as organic N forms (i.e., amino acids, peptides, and proteins) from the soil (Näsholm et al., 2009). Free amino acids generally only account for a small fraction of organic N pool of soil solution whereas peptide- and protein-bound amino acids may contribute to most of the soil organic N pool (Farrell et al., 2011; Hill et al., 2012; Warren, 2014).

Although the ecological significance of organic N uptake for plant N nutrition is still a matter of discussion, several lines of evidence suggest that plants inhabiting ecosystems characterized by slow N mineralization rates may, to a significant degree, rely on organic N forms for growth (Lipson and Näsholm, 2001).

To make the picture more complex, many plant species form intimate symbioses with mycorrhizal fungi, which constitute a large proportion of the microbial biomass in many soils and may play a crucial role in the N nutrition of their host plants (Girlanda et al., 2007; Fitter et al., 2011).

Mycorrhizal fungi are also known to use different N sources, depending on specific biochemical, physiological, and ecological features of the fungus involved (Girlanda et al., 2007). Acquisition of organic N has traditionally been associated with ectomycorrhizal fungi, which are localized in the upper, organic matter-enriched soil horizons. And, indeed, some ectomycorrhizal fungi have been shown to possess proteolytic capabilities and organic N uptake systems (Wipf et al., 2002; Guidot et al., 2005; Benjdia et al., 2006; Shah et al., 2013).

Arbuscular mycorrhizal (AM) fungi, which form relationships with the majority of plant families in most ecosystems, improve the mineral nutrition of their hosts *via* an efficient uptake of mineral nutrients from the soil. AM fungi could also be involved in the acquisition of organic and inorganic N (Cappellazzo et al., 2008; Lanfranco et al., 2011; Pérez-Tienda et al., 2012). Arbuscule-containing cells are thought to be the main site for such a nutrient exchange (Bonfante and Genre, 2010). Arbuscules are highly branched fungal structures that develop inside a living cortex cell: each fungal branch is surrounded by a proliferating plant plasma membrane called the periarbuscular membrane.

It has been shown that organic N uptake is greatly enhanced by AM colonization (Cliquet et al., 1997; Hawkins et al., 2000) and that AM symbiosis could both enhance the decomposition of N and increase N capture from organic patches (Hodge et al., 2001). The uptake of exogenously supplied Arg has also been observed in the extraradical mycelium (ERM) grown in *in vitro* cultures (Govindarajulu et al., 2005; Fellbaum et al., 2012). Apart from the capability of taking up amino acids, there is increasing evidence that AM fungi could increase N capture from more complex organic material (Hodge et al., 2001; Leigh et al., 2009; Whiteside et al., 2013).

N uptake requires the activity of transporters that transfer the N compounds across cellular membranes. Regarding organic N, the only transporter so far described in AM fungi is *GmosAAP1*, an amino acid permease from *Funneliformis mosseae* (Cappellazzo et al., 2008) which may play a role in the first steps of amino acid uptake from the soil. No data are so far available for peptide transporters.

At least four distinct plasma membrane, proton-coupled peptide transport systems have been described in fungi, and in other eukaryotic organisms. Transporters belonging to PTR, DAL5 and FOT families transport di- and tripeptides, while those belonging to OPT family transport longer tetra- and pentapeptides (Hauser et al., 2001; Homann et al., 2005; Reuß and Morschhäuser, 2006; Damon et al., 2011; Hartmann et al., 2011; Dunkel et al., 2013). In *Saccharomyces cerevisiae*, *PTR2* is the only member of the PTR family. Mutants lacking *PTR2* were found to have lost the ability to utilize numerous, but not all, di- and tripeptides as a source of required amino acids (Perry et al., 1994). More recent studies showed that the allantoate/ureidosuccinate permease *DAL5*, contributes to the uptake of the di- and tripeptides that a *ptr2Δ* mutant continued to assimilate (Homann et al., 2005; Cai et al., 2007).

Several PTR members have also been identified in plants and exhibit various functions (Dietrich et al., 2004; Karim et al., 2005; Komarova et al., 2008). For example, in *Arabidospis thaliana* AtPTR5 facilitates peptide transport into germinating and possibly maturing pollen, ovules, and seeds while AtPTR1 has a role in uptake of peptides by roots (Komarova et al., 2008).

In this study we performed the first functional characterization of a putative dipeptide transporter *RiPTR2* from the AM fungus *R. irregularis* which was previously shown to be differentially expressed between mycorrhizal roots and ERM (Tisserant et al., 2012). We further addressed its regulation pattern in intra- and extraradical fungal structures challenged with different N sources in order to understand its potential role in the fungal nutrition and symbiotic interaction.

## Materials and methods

### Biological materials, growth conditions, and treatments

*Rhizophagus irregularis* (Syn. *Glomus intraradices*, DAOM 197198; Krüger et al., 2012) inoculum for seedlings and root organ cultures (ROCs) mycorrhization, was produced through *in vitro* monoxenic cultures. These were established in bi-compartmental Petri dishes with a watertight plastic wall separating the root compartment (RC) from the hyphal compartment (HC) (Fortin et al., 2002). The RC was filled with 25 ml of solid M minimal medium and the HC with 25 ml of solid M medium lacking sugar (M-C). Cultures were started by placing an explant of *Agrobacterium rhizogenes* transformed-chicory (*Cichorium intybus*) roots colonized with the AM fungus in the RC. Once the mycelium of *R. irregularis* had grown over the plastic wall and completely filled the HC compartment, the medium was dissolved with sterile 10 mM citrate buffer, pH 6.0. Spores were then collected and used for plant inoculation.

To obtain the ERM, when the fungus profusely colonized the HC, its content was removed, and the HC was filled with 15 ml liquid M-C medium containing either 3.2 mM (100% N) or 0.8 mM (25% N) KNO^−^_3_. The mycelium was allowed to colonize this medium over the subsequent 2 weeks. Petri dishes were examined regularly and roots were trimmed as required to prevent crossing into the HC.

For the dipeptide treatments, ERM was grown for 2 weeks in liquid M medium (100% N as KNO^−^_3_). At this point, the medium of the HC was removed and replaced by fresh liquid M-C medium containing as nitrogen sources 3.2 mM nitrate, 3.2 mM nitrate and 10 mM Ala-Leu, 10 mM Ala-Leu or no N. ERM was harvested after 24 h.

In both experiments, ERM was collected with tweezers, rinsed with sterilized water, dried with sterilized filter paper, immediately frozen in liquid N and stored at −80°C until used.

To obtain seedlings colonized by *R. irregularis*, the Millipore sandwich method (Giovannetti et al., 1993) was used. Seeds of *Medicago truncatula* Gaertn cv Jemalong were first scarified using sandpaper P180–200, sterilized with 5% commercial bleach for 3 min and rinsed three times for 10 min with sterile distilled water. Germination was induced under sterile conditions in 0.6% agar/water, incubated for 5 days in the dark (25°C) and then exposed at the light for 4 days. Plants were watered with a modified Long-Ashton (LA) solution containing 3.2 μM Na_2_HPO_4_·12H_2_O and 0.5 mM NaNO_3_ nitrate as P and N sources, respectively (Hewitt, 1966) and were grown in a growth chamber under 14 h light (24°C)/10 h dark (20°C) regime. Plants were harvested 60 days post-inoculation (dpi).

For the dipeptide treatment, in a first experiment, *M. truncatula* mycorrhizal roots were obtained in pot cultures watered with a LA solution containing 1 mM nitrate. Two months after inoculation, mycorrhizal roots were treated for 24 h in hydroponic conditions with a LA solution containing 10 mM Ala-Leu or 1 mM nitrate or no N. In a second experiment *M. truncatula* mycorrhizal roots were grown in the sandwich system as described above for 60 days with a modified LA solution containing 2.5 mM Ala-Leu and 0.25 mM nitrate as N sources.

For mycorrhizal plants, only portions of the root system showing extraradical fungal structures were collected under a stereomicroscope. The colonization level was assessed according to Trouvelot et al. (1986). For the molecular analyses, roots were immediately frozen in liquid nitrogen and stored at −80°C.

### Sequence analyses

Fungal protein sequences homologous to PTR2 transporters were identified by BLASTp searches within the Mycocosm database (Grigoriev et al., 2014). Prediction of trans-membrane domains was performed using TMHMM (http://www.cbs.dtu.dk/services/TMHMM-2.0/), SOSUI (http://harrier.nagahama-i-bio.ac.jp/sosui/), HMMTOP (http://www.enzim.hu/hmmtop/) and TMpred (http://www.ch.embnet.org/software/TMPRED_form.html) programs.

Amino acid sequences were aligned using MUSCLE (Edgar, 2004) and their phylogenetic links inferred by using the Maximum Likelihood method based on the JTT matrix-based model (Jones et al., 1992) as implemented in MEGA5 (Tamura et al., 2011).

### Yeast complementation assays

The coding region of *RiPTR2* was amplified from *R. irregularis* cDNAs by PCR using Phusion DNA-Polymerase (Finnzymes, Espoo, Finland) and the two following oligonucleotides containing a *Not*I restriction site: forward primer, 5′-TGACATTGCGGCCGCATAATGGAAGGACACATTCAA-3′; reverse primer, 5′-ACTTCGAGCGGCCGCTGTGACTATTCTTCGGATTTA-3′. The PCR product was cloned into *Not*I sites downstream of the *S. cerevisiae* constitutive PGK1 promoter in the pFL61 *E. coli*-yeast shuttle vector (Minet et al., 1992). Recombinant (pRiPTR2) and empty vector (EV) were used to transform W303 (MAT**a** *ura3 can1–100*) yeast mutants deleted of only one or of its two known dipeptide transporter genes, *PTR2* and *DAL5* (Homann et al., 2005).

Yeast transformation was performed using standard protocols (Rose et al., 1990) and yeast transformants propagating either the EV or the pRiPTR2 recombinant vector were initially selected on a yeast nitrogen base minimal medium lacking uracil. Single colonies of transformants were grown over night in 5 ml of NH^+^_4_-containing yeast carbon base (YCB) liquid medium. Once the OD_600 nm_ reached 0.9, serial dilutions (1:10–1:100–1:1000–1:10,000) in sterile water were prepared and 5 ml plated on YCB solid medium containing H_2_0 (negative control), NH^+^_4_(positive control), Ala-Tyr, Tyr-Ala or Ala-Leu (0.25 mM each). Plates were incubated at 30°C for 4 days and photographed.

### Nucleic acid extraction and RT-qPCR assays

Total genomic DNA was extracted from *R. irregularis* extraradical structures and *M. truncatula* and *C. intybus* roots using the DNeasy Plant Mini Kit (Qiagen), according to the manufacturer's instructions.

Total RNA was isolated from about 100 mg of seedling roots and 20 mg ERM using the RNeasy Plant Mini Kit (Qiagen). Samples were treated with TURBO™ DNase (Ambion) according to the manufacturer's instructions. The RNA samples were routinely checked for DNA contamination by RT-PCR analysis, using primers MtTefF 5′-AAGCTAGGAGGTATTGACAAG-3′ and MtTefR 5′-ACTGTGCAGTAGTACTTGGTG-3′ for *MtTEF* or RiTef-f 5′-GCTATTTTGATCATTGCCGCC-3′ and RiTef-r 5′-TCATTAAAACGTTCTTCCGACC-3′ for *RiTEF* (Gonzàlez-Guerrero et al., 2010) and the One-Step RT-PCR kit (Qiagen). The *MtPT4* phosphate transporter gene was amplified with MtPT4F (5′-TCGCGCGCCATGTTTGTTGT-3′) and MtPT4R (5′-CGCAAGAAGAATGTTAGCCC-3′) primers (Zocco et al., 2011) and the *RiPTR2* peptide transporter with RiPTR2F (5′-GGCTATATTCTTAACGATGTCG-3′) and RiPTR2R (5′-CGACCTGTTCTTCTTCCTCTT-3′) primers. Conventional PCR assays on plant genomic DNA excluded any cross-hybridization of *RiPTR2* specific primers.

For single-strand cDNA synthesis about 500 ng of total RNA were denatured at 65°C for 5 min and then reverse-transcribed at 25°C for 10 min, 42°C for 50 min and 70° for 15 min in a final volume of 20 μl containing 10 μM random hexamers, 0.5 mM dNTPs, 4 μl 5× buffer, 2 μl 0.1 M DTT, and 1 μl Super-ScriptII (Invitrogen).

qRT-PCR experiments were carried out in a final volume of 20 μl containing 10 μl of iTaq™ Universal SYBR® Green Supermix (Bio-Rad), 1 μl of 3 μM specific primers, and about 20 ng of cDNA. Samples were run in the iCycler iQ apparatus (Bio-Rad) using the following program: 10 min pre-incubation at 95°C, followed by 40 cycles of 15 s at 95°C, and 1 min at 60°C. Each amplification was followed by melting curve analysis (60–94°C) with a heating rate of 0.5°C every 15 s. All reactions were performed with three technical replicates and only Ct values with a standard deviation that did not exceed 0.3 were considered. The comparative threshold cycle method (Rasmussen, 2001) was used to calculate relative expression levels using the plant *MtTEF* or the fungal *RiTEF* as reference genes for plant and fungal genes, respectively. The analyses were performed on three independent biological replicates. Statistical tests were carried out through one-way analysis of variance (One-Way ANOVA) and Tukey's *post-hoc* test, using a probability level of *p* < 0.05. All statistical analyses were performed using the PAST statistical package (version 2.16; Hammer et al., 2001).

### Semi-quantitative RT-PCR on laser microdissected cells

*M. truncatula* roots colonized by *R. irregularis* obtained with the millipore sandwich system, as described above, were cut into 5–10 mm-long pieces, treated with ethanol and glacial acetic acid (3:1) under vacuum for 30 min and then placed at 4°C overnight. Roots were subsequently dehydrated in a graded series of ethanol (50%–70%–90% in sterilized water and 100% twice) followed by Neoclear (twice) with each step on ice for 30 min. Neoclear was gradually replaced with paraffin (Paraplast Plus; Sigma-Aldrich, St. Louis) according to Pérez-Tienda et al. (2011). A Leica AS LMD system (Leica Microsystem, Inc.) was used to collect colonized cortical cells from paraffin root sections as described by Balestrini et al. (2007).

RNA was extracted from dissected cells using the PicoPure kit protocol (Arcturus Engineering). A DNAse treatment was performed using an RNA-free DNase Set (Qiagen) in a Pico Pure column, according to the manufacturer's instructions and RNAs were eluted in 21 μl of sterile water.

All RT-PCR assays were carried out using the One Step RT-PCR kit (Qiagen). DNA contaminations were assessed using the *MtTEF* primers described above. Reactions with *RiPTR2* or *MtPT4* specific primers were carried out in a final volume of 10 μl containing 2 μl of 5× buffer, 0.4 μl of 10 mM dNTPs, 1 μl of each primer 10 mM, 0.2 μl of One Step RT-PCR enzyme mix, and 1 μl of a total RNA diluted 1:1. The samples were incubated for 30 min at 50°C, followed by 15 min incubation at 95°C. Amplification reactions were run for 40 cycles of 94°C for 30 s, 60°C for 30 s, and 72°C for 40 s. RT-PCR experiments were conducted on two different biological replicates of 1500–2000 microdissected cells each.

## Results

With the aim to characterize fungal genes involved in fungal/plant nutrient exchange and, possibly, in the functioning of arbuscules, the key structures of the AM symbiosis, we exploited transcriptomics data (Tisserant et al., 2012) generated for the AM fungus *R. irregularis* (Syn. *G. intraradices*; Krüger et al., 2012). We focused our attention on an EST (contig step3_c3279 of MIRA v2 assembly) that, from the microarray data, showed a 33 fold up-regulation in the intraradical mycelium (IRM) compared to the ERM (Tisserant et al., 2012). The sequence, containing a full length ORF of 547 amino acids, had been annotated as an oligopeptide transporter. BLAST searches indicated a similarity to fungal Major Facilitator Superfamily (MFS) di/tripeptide transporters. In particular, the sequence possesses the PTR2[pfam00854] conserved domain that characterizes the proton-dependent oligopeptide transporters (PTR, T.C.2.A.17) family. For this reason, it was called *RiPTR2*.

Recent releases of the complete genome sequence of *R. irregularis* (Tisserant et al., 2013; Lin et al., 2014) allowed us to assess that *RiPTR2* is a single copy gene containing four introns (Figure S1). Twelve transmembrane domains were predicted for RiPTR2 amino acid sequence (Figures S1,S2) using HMMTOP, TMHMM, SOSUI, and TMPRED programs. This structure is similar to that of the well characterized PTR2 sequence from *S. cerevisiae* which shows 40.8% identity at the amino acid level to RiPTR2.

Two putative homologs were found within an extensive transcripts dataset of another AM fungus, *Gigaspora margarita* (Salvioli et al., in preparation), which is phylogenetically distant from *R. irregularis*. The two deduced amino acid sequences, named GmarPTR2A and GmarPTR2B show 56.2 and 51.7% identity to RiPTR2, respectively.

**Figure 1:**
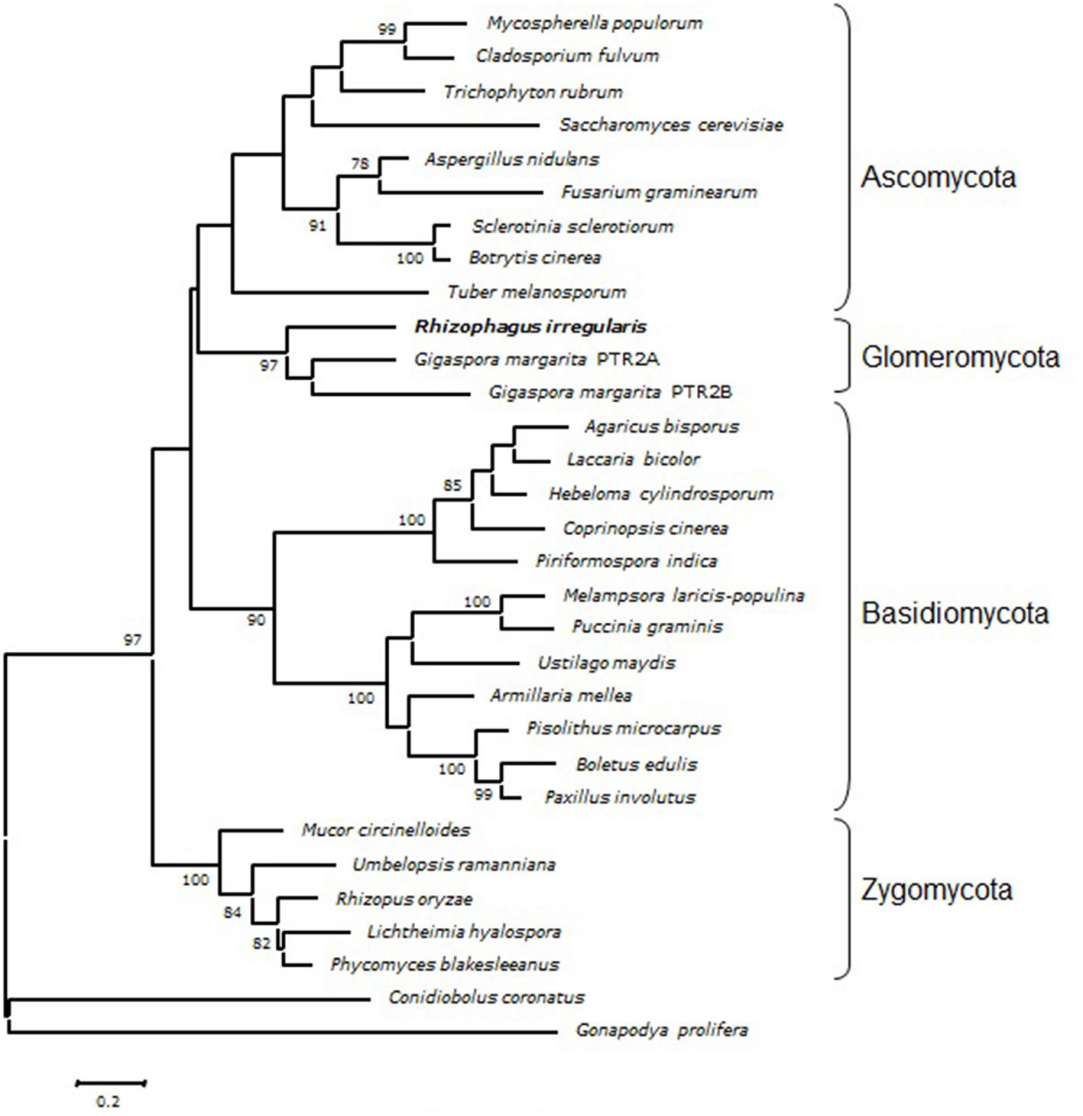
**Phylogenetic position of RiPTR2 protein sequence in comparison with homologous sequences from selected species representative of the major fungal phyla (Table S1)**. The evolutionary history was inferred by using the Maximum Likelihood method based on the JTT matrix-based model as implemented in MEGA5. The percentage of trees higher than 75% in which the associated taxa clustered together is shown next to the branches. The tree is drawn to scale, with branch lengths measured in the number of substitutions per site.

A phylogenetic analysis was carried out by Maximum Likelihood method using PTR2 sequences from a number of fungi representative of distinct taxonomic groups retrieved from the MycoCosm (Grigoriev et al., 2014) genomic database (Figure 1; Table S1). RiPTR2 grouped with the two *G. margarita* sequences to form a well-supported sequence cluster clearly separated from sequences from Ascomycota, Basidiomycota and Zygomycota species.

### RiPTR2 encodes a functional dipeptide transporter

To verify whether *RiPTR2* encodes a functional peptide transporter, it was expressed in *S. cerevisiae* W303 mutant strains deleted of either one or both of its two endogenous di/tripeptide transporter genes, *PTR2* and *DAL5*. Both single (*ptr2Δ* or *dal5Δ* or double (*ptr2Δ dal5Δ*) mutant strains are unable to use many dipeptides as N sources (Homann et al., 2005).

*RiPTR2*-expressing yeast transformants were plated onto selective media, containing one of three different dipeptides as N sources; Ala-Tyr, Tyr-Ala or Ala-Leu. Tyr-Ala is known to be a substrate for the yeast Ptr2p but not for Dal5p, while Ala-Leu is used by Dal5p but not by Ptr2p, and Ala-Tyr is neither used by Ptr2p nor by Dal5p in the W303 background (Homann et al., 2005). In addition, H_2_0 and NH^+^_4_ were used as negative and positive control, respectively.

**Figure 2:**
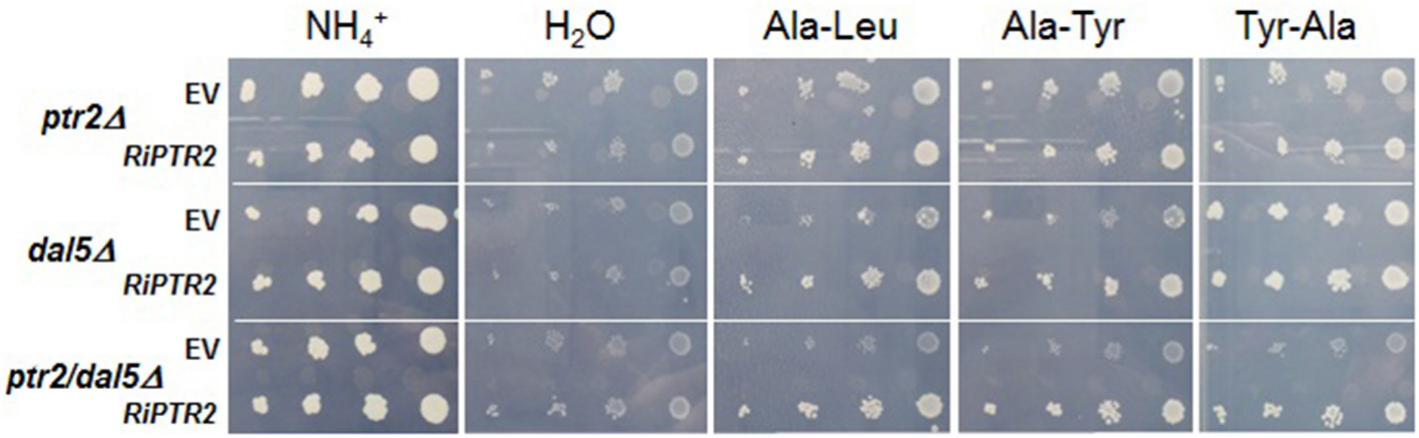
***RiPTR2* complements growth defects of *ptr2Δ*, *dal5Δ* or *ptr2Δ dal5Δ* yeast transporter mutants on dipeptides**. Serial dilutions (1:10–1:100–1:1,000–1:10,000 from left to right) of cultured strains transformed with RiPTR2 or the empty vector (EV) were plated on YCB solid medium containing H_2_0 (negative control), NH^+^_4_ (positive control), 0.25 mM Ala-Tyr, Tyr-Ala or Ala-Leu. Plates were incubated at 30°C for 4 days.

All transformants (empty or recombinant vector) were unable to grow in N-free medium (H_2_O) although a background growth could be observed probably due to residual traces of N compounds present in the agar. As expected, all transformants were able to grow in the NH^+^_4_-containing medium, Interestingly, *ptr2Δ dal5Δ* cells expressing RiPTR2 grew on Ala-Leu, Ala-Tyr and also Tyr-Ala, whereas no growth was observed for *ptr2Δ dal5Δ* cells transformed with the EV, indicating that RiPTR2 is able to transport these three dipeptides (Figure 2).

Ala-Leu and Ala-Tyr transport in the *dal5Δ* single mutant expressing *RiPTR2* was also evident (Figure 2). Results obtained with *ptr2Δ* single mutant were difficult to interpret since this mutant, transformed with the EV, showed a high background growth on all three dipeptides. However, RiPTR2 transformants clearly diplayed an increased growth on all three dipeptides.

Taken as a whole these results demonstrated that *RiPTR2* codes for a functional dipeptide transporter.

### RiPTR2 expression profiles

**Figure 3:**
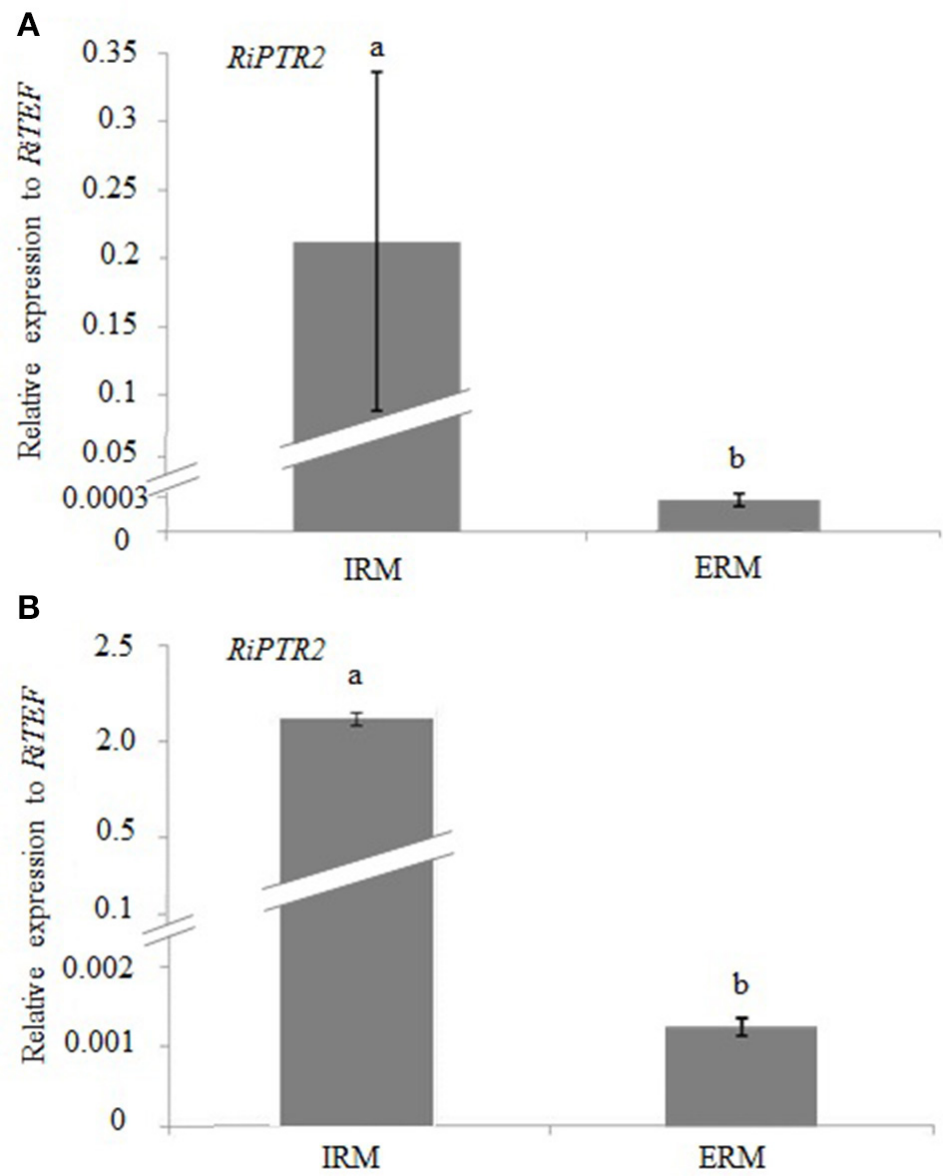
**Relative expression of *RiPTR2* assessed by qRT-PCR in intraradical mycelium (IRM) and extraradical mycelium (ERM) from mycorrhizal roots of *M. truncatula* grown in the sandwich system (A) and mycorrhizal roots of *C. intybus* grown in monoaxenic culture (B)**. Data for each condition are presented as mean ± standard deviation. Different letters indicate statistically significant difference (*p* < 0.05, ANOVA).

We first compared the expression profile of *RiPTR2* in intraradical (IRM) and extraradical (ERM) mycelia by quantitative RT-PCR from *M. truncatula* plants colonized by *R. irregularis* grown in 0.5 mM nitrate in the sandwich system. RNA extractions were performed on ERM and on roots fragments from which the ERM was carefully removed to produce the IRM sample. Samples were normalized with the *RiTEF* housekeeping gene. A strong *RiPTR2* expression was observed in the IRM while *RiPTR2* transcripts were less abundant, and almost barely detected, in the ERM (Figure 3A). A similar result was obtained considering mycorrhizal roots of *C. intybus* devoided of ERM, grown in the root compartment and ERM developed in the hyphal compartment of the same ROC (Figure 3B). These results thus confirmed the strong expression in the IRM as observed in the microarray data (Tisserant et al., 2012).

**Figure 4:**
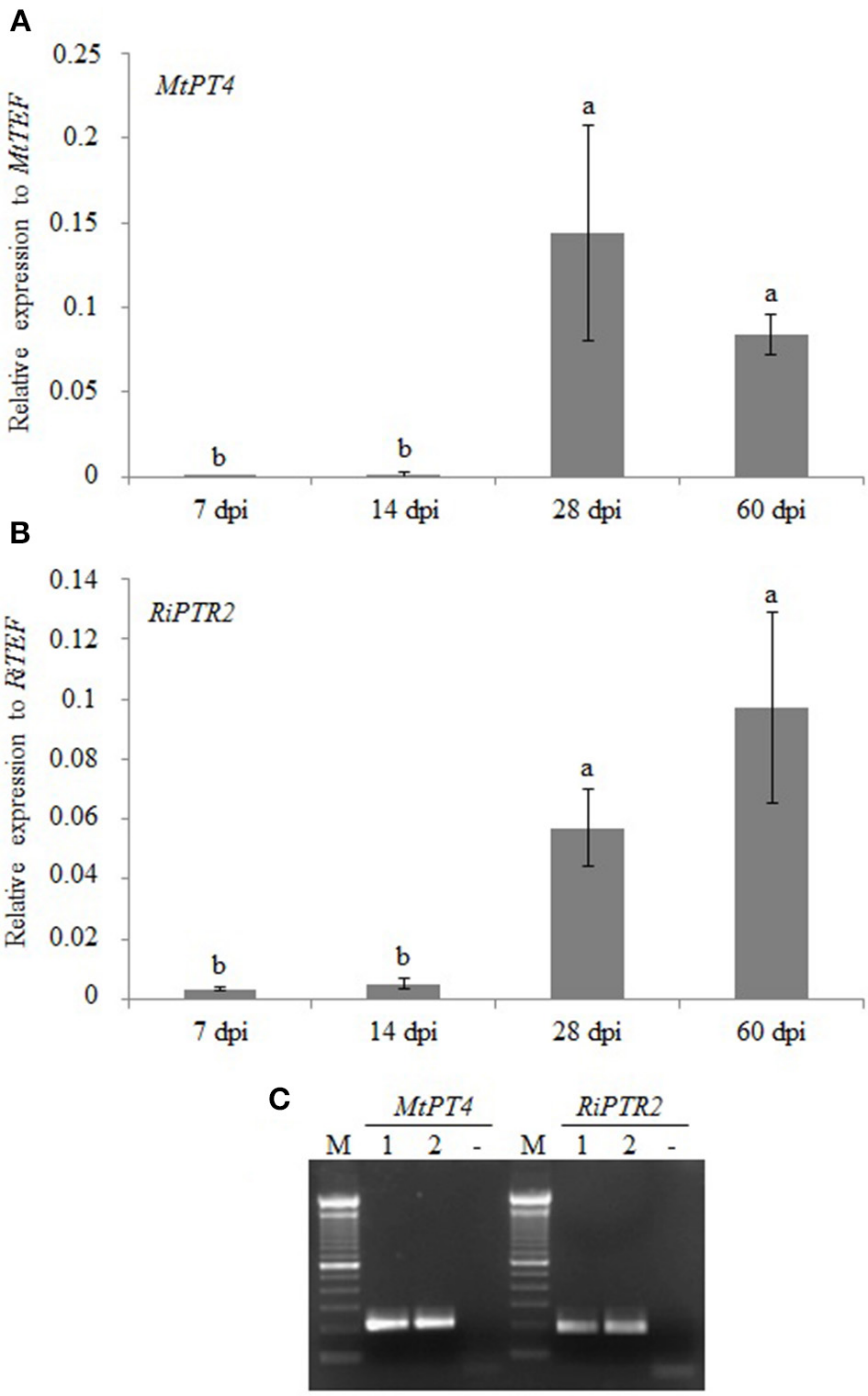
**Relative expression of *MtPT4* (A) and *RiPTR2* (B) assessed by qRT-PCR in a time course experiment of root colonization at 7, 14, 28 and 60 days post-inoculation (dpi)**. Data for each condition are presented as mean ± standard deviation. Different letters indicate statistically significant difference (*p* < 0.05, ANOVA). **(C)** Gel electrophoresis of RT-PCR products obtained from two independent samples (1, 2) of RNA from laser-microdissected arbuscule-containing cells using primers specific for *MtPT4* or *RiPTR2*. No RNA sample (-); M: 100 bp (Invitrogen).

We also performed a time course experiment in the sandwich system to determine *RiPTR2* transcript abundance at different times (7, 14, 28, and 60 days) post-inoculation (dpi) of *M. truncatula* plants. Morphological analyses of roots showed almost no fungal structures at 7 or 14 dpi, while mycorrhization frequency increased from 28 to 60 dpi. Arbuscules were visible starting from 28 dpi and were slightly less abundant at 60 dpi than at 28 dpi (Figure S3). Since in the ERM *RiPTR2* is expressed at negligible levels, gene expression was evaluated in whole mycorrhizal roots, without making a distinction between IRM and ERM. *RiPTR2* mRNA abundance increased in parallel to the development of the intraradical phase as demonstrated by morphological data and the parallel mRNA accumulation of *MtPT4*, the *M. truncatula* phosphate transporter-encoding gene which is considered a molecular marker of arbuscule-containing cells (Harrison et al., 2002; Figures 4A,B).

The laser microdissection technique was used to specifically obtain RNA from *M. truncatula* arbusculated cells. The authenticity of the samples was verified using *MtPT4* specific primers. *RiPTR2* mRNA was detected in the two independent samples analyzed, indicating that, under these conditions, *in planta RiPTR2* expression occurred in arbuscules (Figure 4C).

We then investigated whether the presence of a dipeptide (Ala-Leu), a candidate substrate of RiPTR2 as indicated by the yeast heterologous expression, could modulate *RiPTR2* expression levels. This was tested in both a short- and in a long-term exposure experiments where we evaluated the *RiPTR2* expression in whole mycorrhizal roots, without making a distinction between IRM and ERM.

**Figure 5:**
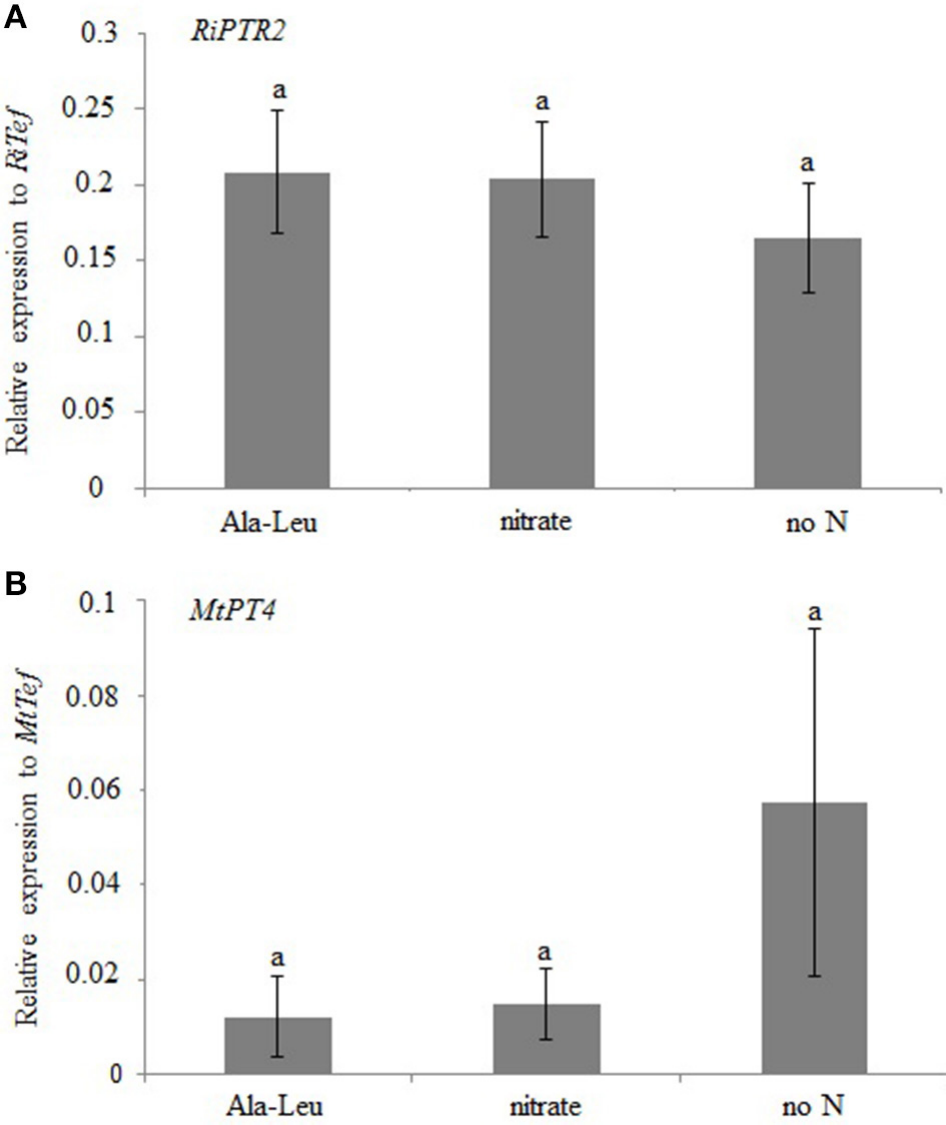
**Relative expression of *RiPTR2* (A) assessed by qRT-PCR in mycorrhizal roots exposed for 24 h to 10 mM Ala-Leu, 1 mM nitrate or no N**. *MtPT4* expression was used as marker of mycorrhization **(B)**. Data for each condition are presented as mean ± standard deviation. Different letters indicate statistically significant difference (*p* < 0.05, ANOVA).

In the first experiment (short term exposure), *M. truncatula* mycorrhizal plants were obtained in pot cultures watered with a Long Ashton (LA) nutrient solution containing 1 mM nitrate. After 2 months mycorrhizal roots were treated for 24 h in hydroponic conditions with a LA solution containing 10 mM Ala-Leu or 1 mM nitrate or no N. No significant difference was observed in *RiPTR2* expression levels among the different treatments (Figure 5A). Plants showed rather similar mycorrhization degrees as revealed by the *MtPT4* expression levels; although no N samples had higher values, they were not statistically different from those of the other two treatments (Figure 5B).

**Figure 6:**
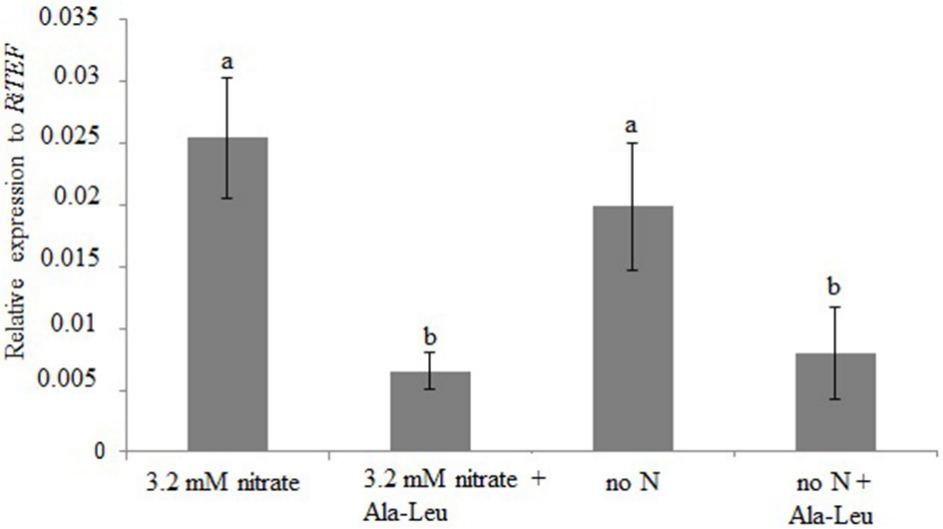
**Relative expression of *RiPTR2* assessed by qRT-PCR in the extraradical mycelium developed in ROC exposed for 24 h to 10 mM Ala-Leu or 3.2 mM nitrate or no N**. Data for each condition are presented as mean ± standard deviation. Different letters indicate statistically significant difference (*p* < 0.05, ANOVA).

We also investigated whether the *RiPTR2* expression in the ERM developed in the ROC system was responsive to 24 h exposure to 10 mM Ala-Leu. As previously observed *RiPTR2* expression in the ERM was extremely low (Figure 3) and this treatment led to an apparent decrease of *RiPTR2* mRNA abundance compared to the ERM kept for 24 h in a medium containing 3.2 mM nitrate or no nitrogen (Figure 6).

**Figure 7:**
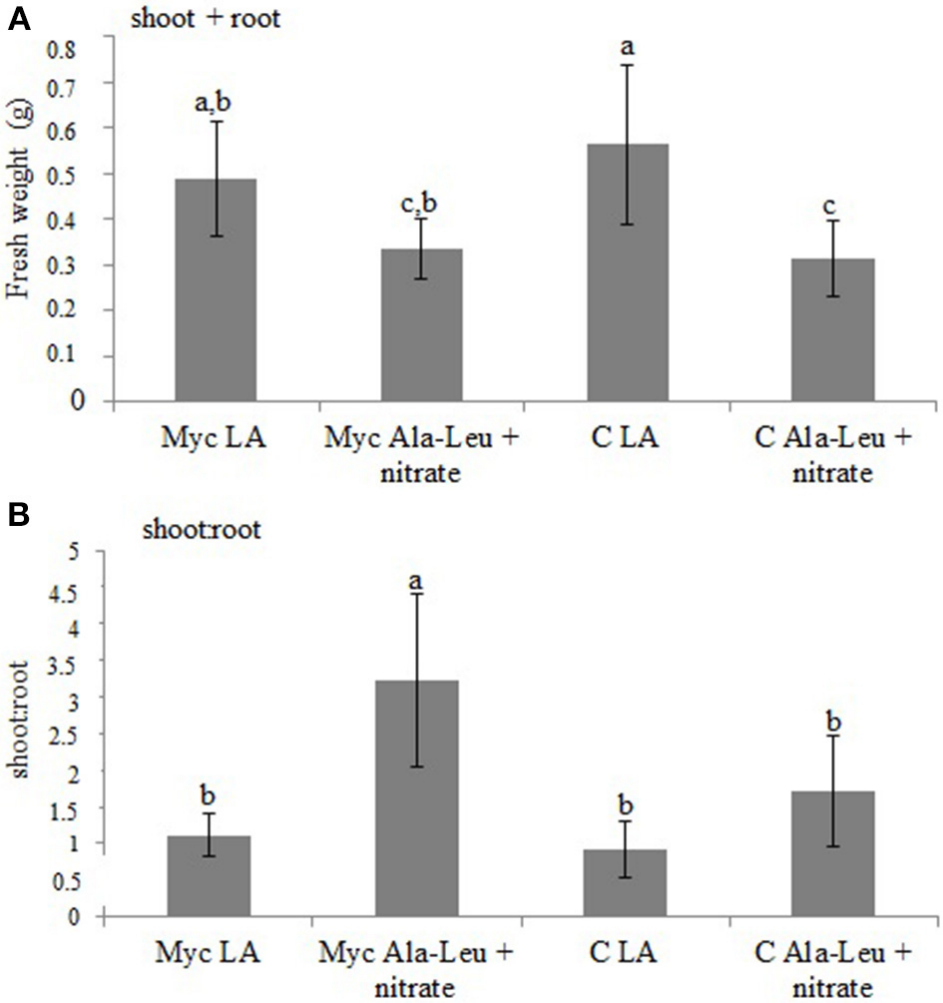
**Total fresh weight (A) and shoot to root ratio (B) of *M. truncatula* plants grown in the sandwich system inoculated (Myc) or not (C) and watered with either 2.5 mM Ala-Leu and 0.25 mM nitrate (Ala-Leu + nitrate) or 0.5 mM nitrate (LA)**. Data for each condition are presented as mean ± standard deviation. Different letters indicate statistically significant difference (*p* < 0.05, ANOVA).

**Figure 8:**
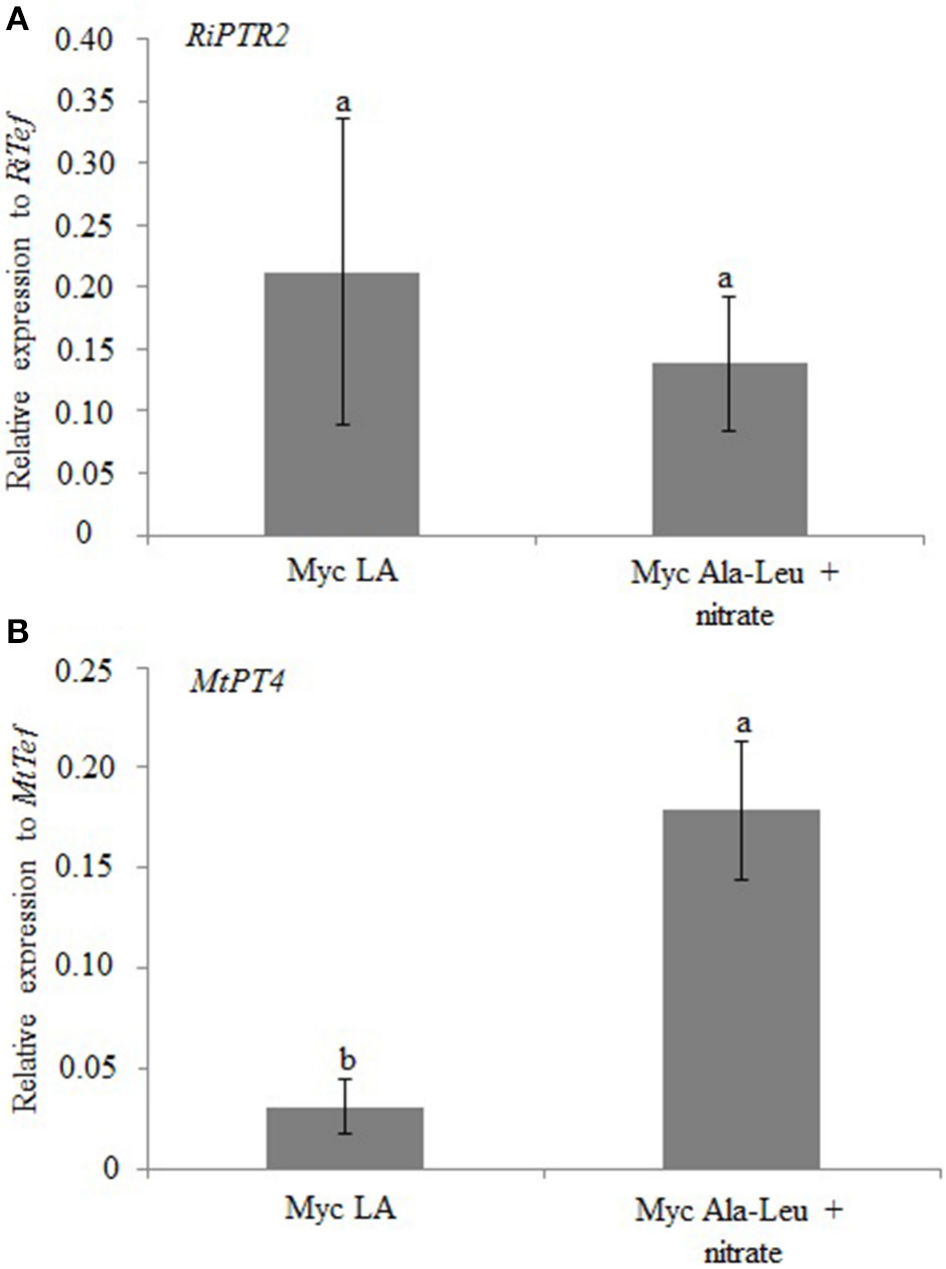
**Relative expression of *RiPTR2* (A) assessed by qRT-PCR in mycorrhizal roots grown in the presence of 0.5 mM nitrate (LA) or 2.5 mM Ala-Leu and 0.25 mM nitrate (Ala-Leu + nitrate)**. *MtPT4* expression was used as marker of mycorrhization **(B)**. Data for each condition are presented as mean ± standard deviation. Different letters indicate statistically significant difference (*p* < 0.05, ANOVA).

In the second experiment (long term exposure), *M. truncatula* mycorrhizal and non-inoculated plants were grown in the sandwich system for 60 days with a nutrient solution containing 2.5 mM Ala-Leu and 0.25 mM nitrate as N sources. Mycorrhizal plants grown in standard nutrient solution, that is 0.5 mM nitrate, were used as controls. Independently from the AM colonization, plants grown in the presence of Ala-Leu + nitrate have total (shoot + root) biomasses lower than plants grown in only nitrate (Figure 7A). Interestingly, when shoot to root ratio is considered, it appears that the mycorrhization has a positive effect on the plant in the presence of Ala-Leu + nitrate, in particular in resource allocation into shoots (Figure 7B). This result suggests that the AM fungal colonization contributes to the more efficient use of N, including dipeptides. This result may also be due to the higher colonization level as revealed by the *MtPT4* expression (Figure 8B). No difference in *RiPTR2* transcript abundance was observed in the two conditions (Figure 8A).

## Discussion

Organic N dominates the N pool of the majority of soils (Schimel and Bennet, 2004). A key concept under revision is that soil microbes have a greater ability to use organic N than plants: indeed there is evidence that plants not only take up organic N (Komarova et al., 2008) but may also compete with microbes for it, although it remains debated how competitive plants are (Kuzyakov and Xu, 2013).

Recent works have shown that, in soils, short peptides represent a greater proportion of N than free amino acids, although there is no detailed information on the availability of individual peptides due to their multiplicity and the lack of a suitable detection system (Hill et al., 2012). Among the peptide uptake systems, the PTR family comprises proton driven symporters which are present in bacteria, fungi, plants and animals. Human PTR transporters have been extensively studied as they are the main route through which the body absorbs and retain dietary protein (Newstead, 2014). Despite their potential importance in the N cycle (Schmidt et al., 2014), still very little is known on PTR from plants or soil fungi.

In this work we have characterized *RiPTR2*, a PTR transporter from the fungus *R. irregularis*, a model species for the ecologically and agriculturally important group of the Glomeromycota. Searches within the two complete genome sequences of *R. irregularis* so far available (Tisserant et al., 2013; Lin et al., 2014) showed that *RiPTR2* is a single copy gene. Interestingly, two putative homologs have been identified within a transcriptome of another AM fungus, *G. margarita*, which belongs to a distinct family of Glomeromycota. PTR2 gene redundancy has also been found in the genomes of several filamentous fungi (Vizcaíno et al., 2006), including the ectomycorrhizal fungus *Hebeloma cylindrosporum* which is, to our knowledge, the only mycorrhizal fungus with characterized PTR2 genes (Benjdia et al., 2006).

The *RiPTR2* sequence was able to complement the growth defects of yeast mutants defective of the two well studied dipeptide transporters. At least in the heterologous system, *RiPTR2* was able to transport Ala-Leu, Ala-Tyr and Tyr-Ala but it is likely that other dipeptides can be transported. Substrate promiscuity is in fact a common feature shared by all peptide uptake systems although the molecular basis of this phenomenon is still an open question (Newstead, 2014). On the other hand, this low substrate specificity has the advantage to avoid restricting uptake to only a subset of the available peptides.

Gene expression data clearly indicated that *RiPTR2* is expressed in the ERM, though at a low level, suggesting a potential role in the uptake of dipeptides from the soil solution. However, at least in the analyzed conditions, mRNA abundance was higher in the intraradical phase. The time course experiment clearly showed an up-regulation of *RiPTR2* during the colonization process and the establishment of mature mycorrhizas. The analysis of laser-microdissected arbuscule-containing cells confirmed that *RiPTR2* is consistently expressed in arbuscules. Since *RiPTR2* mRNA abundance does not perfectly match *MtPT4* mRNA accumulation (at 28 or 60 dpi) we suggest that *RiPTR2* may be expressed not only in arbuscules but also in intercellular hyphae. Evidence that nutrient exchange goes beyond the interface of arbusculated cells and could involve intercellular hyphae as well has been obtained from the expression profile of a fungal monosaccharide transporter (Helber et al., 2011).

A high expression level in the intraradical phase, including arbuscules, where the flux is commonly expected to be directed toward the host plant cells, is puzzling. However, it is worth noticing that additional transporters, a phosphate one (Balestrini et al., 2007; Tisserant et al., 2012; Fiorilli et al., 2013) and an ammonium one (Pérez-Tienda et al., 2011) from the fungal symbiont were found to be consistently expressed in arbuscules. Dealing with organic N, *R. irregularis* transcriptomic data also showed the up-regulation of a putative amino acid transporter (contig Glomus_c20826) in the IRM vs. the ERM (Tisserant et al., 2012). These findings suggest that the fungus may reabsorb nutrients released in the periarbuscular space and thus exerts a control over the amount of nutrients delivered to the host. The interface compartment that surrounds the arbuscules is considered acidic (Guttenberger, 2000) and provides a gradient in the electrochemical potential used for the energization of uptake processes. It could be an ideal environment for PTR2 proteins, since they work as proton-coupled transporters and usually show the highest transport activity at acidic pH (Benjdia et al., 2006; Komarova et al., 2008). Interestingly, two *M. truncatula* PTR genes were also described as mycorrhiza-responsive and their transcripts detected in arbusculated cells (Gomez et al., 2009). We confirmed the up-regulation of these two *M. truncatula* genes in mycorrhizal roots compared to control roots in the long term experiment (data not shown).

*RiPTR2* gene expression is responsive to the Ala-Leu dipeptide, a possible substrate. We observed a down-regulation of *RiPTR2* in the ERM grown in ROC after 24 h exposure to 10 mM Ala-Leu. A down-regulation was also found for a dipeptide transporter of the ectomycorrhizal fungus *H. cylindrosporum* after exposure to millimolar dipeptide concentrations (Benjdia et al., 2006). By contrast, *RiPTR2* expression in whole mycorrhizal roots did not change after 24 h exposure to 10 mM Ala-Leu. Since these expression values are likely to be mainly representative of the intraradical phase, we can argue that 24 h are not sufficient to perceive the dipeptide, and that the intraradical *RiPTR2* expression is probably also regulated by the plant endogeneous N status which may not be affected by the short term exposure.

Interestingly, our data from the long term experiment suggest that the AM fungal colonization contributes to the more efficient use of N when nitrate is present in limiting condition and together with a dipeptide.

A part from yeasts (*S. cerevisiae, Schizosaccharomyces pombe*) where *PTR2* expression is induced by either the presence of amino acids or dipeptides (Kitamura et al., 2012; Ljungdahl and Daignan-Fornier, 2012), very little is known on the regulation of *PTR* genes in filamentous fungi, with the exception of *H. cylindrosporum* (Benjdia et al., 2006). A sequence showing high similarity to *PTR* genes was recently found to be expressed during growth on protein-containing substrates in the ectomycorrhizal fungus *P. involutus* (Shah et al., 2013). A *PTR2* gene from the biocontrol agent *Trichoderma harzianum* was up-regulated when the fungus interacted with the plant pathogen *Botrytis cinerea* (Vizcaíno et al., 2006) and during growth on cell wall of *Fusarium solani* (Vieira et al., 2013) indicating that *PTR* genes are involved in the mycoparasitic process. Furthermore, since Glomeromycota are very distantly related to commonly studied fungal species, we may anticipate that regulation of their *PTR* genes can respond to different environmental clues.

In their whole our results show that *RiPTR2* is expressed in the ERM, the fungus-soil interface, suggesting a role in the uptake of organic N from soil; however, a stronger expression is consistently observed in the *in planta* phase, including arbuscules, pointing to a function in the mobilization of organic N in mycorrhizal roots. Further investigations, not only on the fungal but also on the plant side, will help to obtain a more comprehensive view of the dipeptide metabolism in the AM symbiosis.

## Author contributions

Valentina Fiorilli and Simone Belmondo carried out the majority of the experiments and drafted the manuscript. Nuria Ferrol and Jacob Pérez-Tienda contributed to the experiments on the ERM grown in monoaxenic cultures. Roland Marmeisse participated in the design of the work and helped with the yeast complementation. Luisa Lanfranco coordinated the project and wrote the manuscript. All authors read and approved the final manuscript.

### Conflict of interest statement

The authors declare that the research was conducted in the absence of any commercial or financial relationships that could be construed as a potential conflict of interest.

## References

[B1] Balestrini R. Gomez-Ariza J. Lanfranco L. Bonfante P. (2007). Laser microdissection reveals that transcripts for five plant and one fungal phosphate transporter genes are contemporaneously present in arbusculated cells. Mol. Plant Microbe Interact. 20, 1055–1062 10.1094/MPMI-20-9-1055 17849708

[B2] Benjdia M. Rikirsch E. Müller T. Morel M. Corratgé C. Zimmermann S. (2006). Peptide uptake in the ectomycorrhizal fungus *Hebeloma cylindrosporum*: characterization of two di- and tripeptide transporters (HcPTR2A and B). New Phytol. 170, 401–410 10.1111/j.1469-8137.2006.01672.x 16608464

[B3] Bonfante P. Genre A. (2010). Mechanisms underlying beneficial plant-fungus interactions in mycorrhizal symbiosis. Nat. Commun. 1, 48 10.1038/ncomms1046 20975705

[B4] Cai H. Hauser M. Naider F. Becker J. M. (2007). Differential regulation and substrate preferences in two peptide transporters of *Saccharomyces cerevisiae*. Eukaryot. Cell 6, 1805–1813 10.1128/EC.00257-06 17693598 PMC2043388

[B5] Cappellazzo G. Lanfranco L. Fitz M. Wipf D. Bonfante P. (2008). Characterization of an amino acid permease from the endomycorrhizal fungus *Glomus mosseae*. Plant Physiol. 147, 429–437 10.1104/pp.108.117820 18344417 PMC2330287

[B6] Cliquet J. B. Murray P. J. Boucaud J. (1997). Effect of the arbuscular mycorrhizal fungus *Glomus fasciculatum* on the uptake of amino nitrogen by *Lolium perenne*. New Phytol. 137, 345–349 10.1046/j.1469-8137.1997.00810.x 33863187

[B7] Damon C. Vallon L. Zimmermann S. Haider M. Z. Galeote V. Dequin S. (2011). A novel fungal family of oligopeptide transporters identified by functional metatranscriptomics of soil eukaryotes. ISME J. 5, 1871–1880 10.1038/ismej.2011.67 21654847 PMC3223307

[B8] Dietrich D. Hammes U. Thor K. Suter-Grotemeyer M. Fluckiger R. Slusarenko A. J. (2004). AtPTR1, a plasma membrane peptide transporter expressed during seed germination and in vascular tissue of *Arabidopsis*. Plant J. 40, 488–499 10.1111/j.1365-313X.2004.02224.x 15500465

[B9] Dunkel N. Hertlein T. Franz R. Reuß O. Sasse C. Schäfer T. (2013). Roles of different peptide transporters in nutrient acquisition in *Candida albicans*. J. Eukaryot. Cell 12, 520–528 10.1128/EC.00008-13 23376942 PMC3623439

[B10] Edgar R. C. (2004). MUSCLE: multiple sequence alignment with high accuracy and high throughput. Nucleic Acids Res. 32, 1792–1797 10.1093/nar/gkh340 15034147 PMC390337

[B11] Farrell M. Hill P. W. Farrar J. F. Bardgett R. D. Jones D. L. (2011). Seasonal variation in soluble soil carbon and nitrogen across a grassland productivity gradient. Soil Biol. Biochem. 43, 835–844 10.1016/j.soilbio.2010.12.022

[B12] Fellbaum C. Gachomo E. W. Beesetty Y. Choudhari S. Strahan G. D. Pfeffer P. E. (2012). Carbon availability triggers fungal nitrogen uptake and transport in arbuscular mycorrhizal symbiosis. Proc. Natl. Acad. Sci. U.S.A. 109, 2666–2671 10.1073/pnas.1118650109 22308426 PMC3289346

[B13] Fiorilli V. Lanfranco L. Bonfante P. (2013). The expression of *GintPT*, the phosphate transporter of *Rhizophagus irregularis*, depends on the symbiotic status and phosphate availability. Planta 237, 1267–1277 10.1007/s00425-013-1842-z 23361889

[B14] Fitter A. H. Helgason T. Hodge A. (2011). Nutritional exchanges in the arbuscular mycorrhizal symbiosis: implications for sustainable agriculture. Fungal Biol. Rev. 25, 68–72 10.1016/j.fbr.2011.01.002

[B15] Fortin J. A. Bécard G. Declerck S. Dalpé Y. St-Arnaud M. Coughlan A. P. (2002). Arbuscular mycorrhiza on root-organ cultures. Can. J. Bot. 80, 1–20, 10.1139/b01-139

[B16] Giovannetti M. Sbrana C. Avio L. Citernesi A. S. Logi C. (1993). Differential hyphal morphogenesis in arbuscular mycorrhizal fungi during pre-infection stages. New Phytol. 125, 587–593 10.1111/j.1469-8137.1993.tb03907.x 33874594

[B17] Girlanda M. Perotto S. Bonfante P. (2007). Mycorrhizal fungi: their habitats and nutritional strategies, in The Mycota IV-Environmental and Microbial Relationships, eds Kubicek C. P. Druzhinina I. S. (Berlin: Springer), 229–256

[B18] Gomez S. K. Javot H. Deewatthanawong P. Torres-Jerez I. Tang Y. Blancaflor E. B. (2009). *Medicago truncatula* and *Glomus intraradices* gene expression in cortical cells harboring arbuscules in the arbuscular mycorrhizal symbiosis. BMC Plant Biol. 9:10 10.1186/1471-2229-9-10 19161626 PMC2649119

[B19] Gonzàlez-Guerrero M. Oger E. Benabdellah K. Azcón-Aguilar C. Lanfranco L. Ferrol N. (2010). Characterization of a CuZn superoxide dismutase gene in the arbuscular mycorrhizal fungus *Glomus intraradices*. Curr. Genet. 56, 265–274 10.1007/s00294-010-0298-y 20379721

[B20] Govindarajulu M. Pfeffer P. E. Jin H. Abubaker J. Douds D. D. Allen J. W. (2005). Nitrogen transfer in the arbuscular mycorrhizal symbiosis. Nature 435, 819–823 10.1038/nature03610 15944705

[B21] Grigoriev I. V. Nikitin R. Haridas S. Kuo A. Ohm R. Otillar R. (2014). MycoCosm portal: gearing up for 1000 fungal genomes. Nucleic Acids Res. 42, D699–D704 10.1093/nar/gkt1183 24297253 PMC3965089

[B22] Guidot A. Verner M.-C. Debaud J. C. Marmeisse R. (2005). Intraspecific variation in use of different organic nitrogen sources by the ectomycorrhizal fungus *Hebeloma cylindrosporum*. Mycorrhiza 15, 167–177 10.1007/s00572-004-0318-1 15322964

[B23] Guttenberger M. (2000). Arbuscules of vesicular-arbuscular mycorrhizal fungi inhabit an acidic compartment within plant roots. Planta 211, 299–304 10.1007/s004250000324 10987547

[B24] Hammer Ø. Harper D. A. T. Ryan P. D. (2001). PAST: paleontological statistics software package for education and data analysis. Palaeont. Elect. 4, 9 Available online at: http://palaeo-electronica.org/2001_1/past/issue1_01.htm

[B25] Harrison M. J. Dewbre G. R. Liu J. (2002). A phosphate transporter from *Medicago truncatula* involved in the acquisition of phosphate released by arbuscular mycorrhizal fungi. Plant Cell 14, 2413–2429 10.1105/tpc.004861 12368495 PMC151226

[B26] Hartmann T. Cairns T. C. Olbermann P. Morschhäuser J. Bignell E. M. Krappmann S. (2011). Oligopeptide transport and regulation of extracellular proteolysis are required for growth of *Aspergillus fumigatus* on complex substrates but not for virulence. Mol. Microbiol. 82, 917–935 10.1111/j.1365-2958.2011.07868.x 22023286

[B27] Hauser M. Narita V. Donhardt A. M. Naider F. Becker J. M. (2001). Multiplicity and regulation of genes encoding peptide transporters in *Saccharomyces cerevisiae*. Mol. Membr. Biol. 18, 105–112 10.1080/09687680010029374 11396605

[B28] Hawkins H. J. Johansen A. George E. (2000). Uptake and transport of organic and inorganic nitrogen by arbuscular mycorrhizal fungi. Plant Soil 226, 275–285 10.1023/A:1026500810385

[B29] Helber N. Wippel N. Schaarschmidt S. Hause B. Requena N. (2011). A versatile monosaccharide transporter that operates in the arbuscular mycorrhizal fungus *Glomus* sp. is crucial for the symbiotic relationship with plants. Plant Cell 23, 3812–3823 10.1105/tpc.111.089813 21972259 PMC3229151

[B30] Hewitt E. J. (1966). Sand and water culture methods used in the study of plant nutrition, in Technical Communication No. 22 (Kent: Commonwealth Agriculture Bureau), 431–432

[B31] Hill P. W. Farrell M. Jones D. L. (2012). Bigger may be better in soil N cycling: does rapid acquisition of small L-peptides by soil microbes dominate fluxes of protein-derived N in soil? Soil Biol. Biochem. 48, 106–112 10.1016/j.soilbio.2012.01.023

[B32] Hodge A. Campbell C. D. Fitter A. H. (2001). An arbuscular mycorrhizal fungus accelerates decomposition and acquires nitrogen directly from organic material. Nature 413, 297–299 10.1038/35095041 11565029

[B33] Homann O. R. Cai H. Becker J. M. Lindquist S. L. (2005). Harnessing natural diversity to probe metabolic pathways. PLoS Genet. 1:e80 10.1371/journal.pgen.0010080 16429164 PMC1342634

[B34] Jones D. T. Taylor W. R. Thornton J. M. (1992). The rapid generation of mutation data matrices from protein sequences. Comput. Appl. Biosci. 8, 275–282 10.1093/bioinformatics/8.3.275 1633570

[B35] Karim S. Lundh D. Holmstrom K. O. Mandal A. Pirhonen M. (2005). Structural and functional characterization of AtPTR3, a stress-induced peptide transporter of *Arabidopsis*. J. Mol. Model. 11, 226–236 10.1007/s00894-005-0257-6 15889294

[B36] Kitamura K. Nakase M. Tohda H. Takegawa K. (2012). The ubiquitin ligase Ubr11 is essential for oligopeptide utilization in the fission yeast *Schizosaccharomyces pombe*. Eukaryot. Cell 11, 302 10.1128/EC.05253-11 22226946 PMC3294451

[B37] Komarova N. Y. Thor K. Gubler A. Meier S. Dietrich D. Weichert A. (2008). AtPTR1 and AtPTR5 transport dipeptides *in planta*. Plant Physiol. 148, 856–869 10.1104/pp.108.123844 18753286 PMC2556804

[B38] Krüger M. Krüger C. Walker C. Stockinger H. Schußler A. (2012). Phylogenetic reference data for systematics and phylotaxonomy of arbuscular mycorrhizal fungi from phylum to species level. New Phytol. 193, 970–984 10.1111/j.1469-8137.2011.03962.x 22150759

[B39] Kuzyakov Y. Xu X. (2013). Competition between roots and microorganisms for nitrogen: mechanism and ecological significance. New Phytol. 198, 656–669 10.1111/nph.12235 23521345

[B40] Lanfranco L. Guether M. Bonfante P. (2011). Arbuscular mycorrhizas and N acquisition by plants, in Ecological Aspects of Nitrogen Metabolism in Plants, eds Polacco J. C. Todd C. D. (Hoboken, NJ: John Wiley & Sons, Inc.), 52–68

[B41] Leigh J. Hodge A. Fitter A. H. (2009). Arbuscular mycorrhizal fungi can transfer substantial amounts of nitrogen to their host plant from organic material. New Phytol. 181, 199–207 10.1111/j.1469-8137.2008.02630.x 18811615

[B42] Lin K. Limpens E. Zhang Z. Ivanov S. Saunders D. G. O. Mu D. (2014). Single nucleus genome sequencing reveals high similarity among nuclei of an endomycorrhizal fungus. PLoS Genet. 10:e1004078 10.1371/journal.pgen.1004078 24415955 PMC3886924

[B43] Lipson D. Näsholm T. (2001). The unexpected versatility of plants: organic nitrogen use and availability in terrestrial ecosystems. Oecologia 128, 305–316 10.1007/s004420100693 24549899

[B44] Ljungdahl P. O. Daignan-Fornier B. (2012). Regulation of amino acid, nucleotide, and phosphate metabolism in *Saccharomyces cerevisiae*. Genetics 190, 885–929 10.1534/genetics.111.133306 22419079 PMC3296254

[B45] Minet M. Dufour M. E. Lacroute F. (1992). Complementation of mutants by *Arabidopsis thaliana* cDNA. Plant J. 32, 417–422 10.1046/j.1365-313X.1992.t01-38-00999.x 1303803

[B46] Näsholm T. Kielland K. Ganeteg U. (2009). Uptake of organic nitrogen by plants. New Phytol. 182, 31–48 10.1111/j.1469-8137.2008.02751.x 19210725

[B47] Newstead S. (2014). Molecular insights into proton coupled peptide transport in the PTR family of oligopeptide transporters. Biochem. Biophys. Acta. [Epub ahead of print]. 10.1016/j.bbagen.2014.05.011 24859687 PMC4331665

[B48] Pérez-Tienda J. Testillano P. S. Balestrini R. Fiorilli V. Azcon-Agiular C. Ferrol N. (2011). GintAMT2, a new member of the ammonium transporter family in the arbuscular mycorrhizal fungus *Glomus intraradices*. Fungal Genet. Biol. 48, 1044–1055 10.1016/j.fgb.2011.08.003 21907817

[B49] Pérez-Tienda J. Valderas A. Camañes G. García-Agustín P. Ferrol N. (2012). Kinetics of NH^+^_4_ uptake by the arbuscular mycorrhizal fungus *Rhizophagus irregularis*. Mycorrhiza 22, 485–491 10.1007/s00572-012-0452-0 22752460

[B50] Perry J. R. Basrai M. A. Steiner H. Y. Naider F. Becker J. M. (1994). Isolation and characterization of a *Saccharomyces cerevisiae* peptide transport gene. Mol. Cell. Biol. 14, 104–115 10.1128/MCB.14.1.104 8264579 PMC358361

[B51] Rasmussen R. (2001). Quantification on the LightCycler instrument, in Rapid Cycle Real-Time PCR: Methods and Applications, eds Meuer S. Wittwer C. Nakagawara K. (Heidelberg: Springer-Verlag), 21–34

[B52] Reuß O. Morschhäuser J. (2006). A family of oligopeptide transporters is required for growth of *Candida albicans* on proteins. Mol. Microbiol. 60, 795–812 10.1111/j.1365-2958.2006.05136.x 16629678

[B53] Rose M. D. Winston F. Hieter P. (1990). Methods in Yeast Genetics: A Laboratory Course Manual. New York, NY: Cold Spring Harbor Laboratory Press

[B54] Schimel J. P. Bennet J. (2004). Nitrogen mineralization: challenges of a changing paradigm. Ecology 85, 591–602 10.1890/03-8002

[B55] Schmidt S. Torgny Näsholm T. Rentsch D. (2014). Organic nitrogen. New Phytol. 203, 29–31 10.1111/nph.12851 24889363

[B56] Shah F. Rineau F. Canbäck B. Johansson T. Tunlid A. (2013). The molecular components of the extracellular protein-degradation pathways of the ectomycorrhizal fungus *Paxillus involutus*. New Phytol. 200, 875–887 10.1111/nph.12425 23902518 PMC4282482

[B57] Tamura K. Peterson D. Peterson N. Stecher G. Nei M. Kumar S. (2011). MEGA5: molecular evolutionary genetics analysis using maximum likelihood, evolutionary distance, and maximum parsimony methods. Mol. Biol. Evol. 28, 2731–2739 10.1093/molbev/msr121 21546353 PMC3203626

[B58] Tisserant E. Kohler A. Dozolme-Seddas P. Balestrini R. Benabdellah K. Colard A. (2012). The transcriptome of the arbuscular mycorrhizal fungus *Glomus intraradices* (DAOM 197198) reveals functional tradeoffs in an obligate symbiont. New Phytol. 193, 755–769 10.1111/j.1469-8137.2011.03948.x 22092242

[B59] Tisserant E. Malbreil M. Kuo A. Kohler A. Symeonidi A. Balestrini R. (2013). Genome of an arbuscular mycorrhizal fungus provides insight into the oldest plant symbiosis. Proc. Natl. Acad. Sci. U.S.A. 110, 20117–20122 10.1073/pnas.1313452110 24277808 PMC3864322

[B60] Trouvelot A. Kough J. L. Gianinazzi-Pearson V. (1986). Mesure du taux de mycorrhization VA d'un système radiculaire. Recherche de méthodes d'estimation ayant une signification fonctionnelle, in Physiological and Genetical Aspects of Mycorrhizae, eds Gianinazzi-Pearson V. Gianinazzi S. (Paris: INRA), 217–221

[B61] Vieira P. M. Coelho A. S. G. Steindorff A. S. de Siqueira S. J. L. Silva R. Ulhoa C. J. (2013). Identification of differentially expressed genes from *Trichoderma harzianum* during growth on cell wall of *Fusarium solani* as a tool for biotechnological application. BMC Genom. 14:177 10.1186/1471-2164-14-177 23497274 PMC3606605

[B62] Vizcaíno J. A. Cardoza R. E. Hauser M. Hermosa R. Rey M. Llobell A. (2006). ThPTR2, a di/tri-peptide transporter gene from *Trichoderma harzianum*. Fung. Genet. Biol. 43, 234–246 10.1016/j.fgb.2005.12.003 16466953

[B63] Warren C. R. (2014). Organic N molecules in the soil solution: what is known, what is unknown and the path forwards. Plant Soil 375, 1–19 10.1007/s11104-013-1939-y

[B64] Whiteside M. D. Garcia M. O. Treseder K. K. (2013). Amino acid uptake in arbuscular mycorrhizal plants. PLoS ONE 7:e47643 10.1371/journal.pone.0047643 23094070 PMC3475604

[B65] Wipf D. Benjdia M. Tegeder M. Frommer W. B. (2002). Characterization of a general amino acid permease from *Hebeloma cylindrosporum*. FEBS Lett. 528, 119–124 10.1016/S0014-5793(02)03271-4 12297290

[B66] Zocco D. Van Aarle I. M. Oger E. Lanfranco L. Declerck S. (2011). Fenpropimorph and fenhexamid impact phosphorus translocation by arbuscular mycorrhizal fungi. Mycorrhiza 21, 363–374 10.1007/s00572-010-0344-0 21085999

