# Supplementary material for: A dipeptide transporter from the arbuscular mycorrhizal fungus Rhizophagus irregularis is upregulated in the intraradical phase
Source: Front Plant Sci. 2014 Sep 3;5:436. doi: 10.3389/fpls.2014.00436 (PMC4153046; doi:10.3389/fpls.2014.00436)
Supplement: Supplementary file 1 [file Table1.PDF]

Table S1

List of sequences retrieved within the MycoCosm database (Grigoriev *et al.*, 2014) and used to contruct the phylogenetic tree shown in Figure 1. The amino acid (aa) sequence length is shown. A: Ascomycota; B: Basidiomycota; Z: Zygomycota.

**Fungus**	**aa**	**ID**
Ustilago maydis (B)	638	jgi|Ustma1|6138|UM06138
Melampsora laricis-populina (B)	615	jgi|Mellp1|33929|e_gw1.6.38.1
Puccinia graminis (B)	630	jgi|Pucgr1|32338|PGTT_11807
Agaricus bisporus (B)	591	jgi|Agabi_varbisH97_2|195900|estExt_fgenesh2_kg.C_140220
Armillaria mellea (B)	591	jgi|Armme1|7508|g4799.t1
Boletus edulis (B)	579	jgi|Boled1|980315|gm1.4766_g
Coprinopsis cinerea (B)	602	jgi|Copci1|17294|fgeneshDR_te_pg.Chr_5_#_135
Hebeloma cylindrosporum (B)	602	jgi|Hebcy2|240729|CE160469_18248
Laccaria bicolor (B)	603	jgi|Lacbi2|301981|Lacbi1.eu2.Lbscf0001g14300
Paxillus involutus (B)	600	jgi|Paxin1|14579|gm1.9150_g
Piriformospora indica (B)	602	jgi|Pirin1|73785|mRNA:PIIN_02982
Pisolithus microcarpus (B)	597	jgi|Pismi1|686968|fgenesh1_kg.194_#_16_#_Locus2678v1rpkm70.54
Trichophyton rubrum (A)	562	gi|327302644|ref|XP_003236014.1|
Aspergillus nidulans (A)	587	gi|67525901|ref|XP_661012.1|
Sclerotinia sclerotiorum (A)	595	gi|156044036|ref|XP_001588574.1|
Fusarium graminearum (A)	596	gi|46115010|ref|XP_383523.1|
Mycosphaerella populorum (A)	608	gi|453084580|gb|EMF12624.1|
Tuber melanosporum (A)	623	jgi|Tubme1|4802|GSTUMT00007938001
Cladosporium fulvum (A)	917	jgi|Clafu1|189944|scf7180000130302_G9272
Botrytis cinerea (A)	595	jgi|Botci1|4875|BC1T_15335
Saccharomyces cerevisiae (A)	601	jgi|Sacce1|3934|YKR093W
Mucor circinelloides (Z)	599	gi|510999607|gb|EPB81219.1|
Lichtheimia hyalospora (Z)	536	jgi|Lichy1|123512|e_gw1.12.135.1
Phycomyces blakesleeanus (Z)	562	jgi|Phybl2|136953|estExt_Genewise1Plus.C_300084
Rhizopus oryzae (Z)	566	jgi|Rhior3|12637|RO3G_10839
Umbelopsis ramanniana (Z)	577	jgi|Umbra1|245200|fgenesh1_kg.35_#_72_#_combest_scaffold_35_75071
Conidiobolus coronatus	485	jgi|Conco1|23097|gw1.31.17.1
Gonapodya prolifera	573	jgi|Ganpr1|53832|fgenesh1_kg.12_#_132_#_Locus33v1rpkm2150.68
