# Supplementary figures and images for: A dipeptide transporter from the arbuscular mycorrhizal fungus Rhizophagus irregularis is upregulated in the intraradical phase
Source: Front Plant Sci. 2014 Sep 3;5:436. doi: 10.3389/fpls.2014.00436 (PMC4153046; doi:10.3389/fpls.2014.00436)

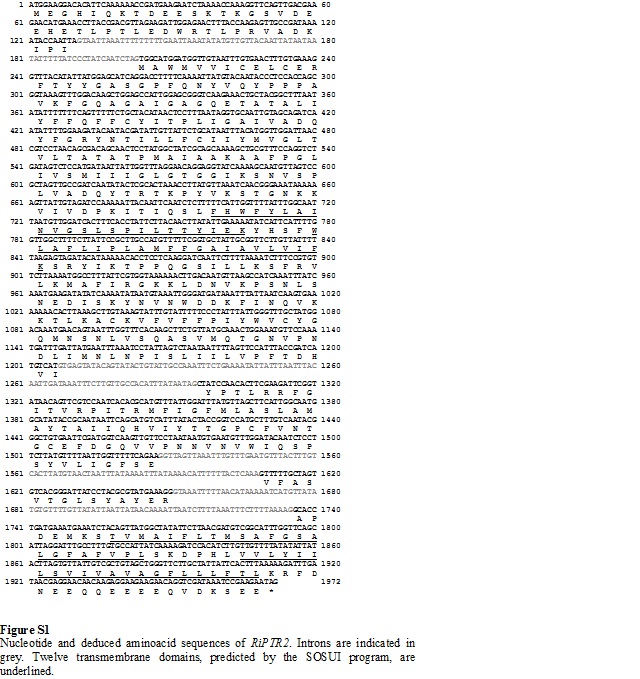

Supplement: Supplementary file 2 [file Image1.JPEG]

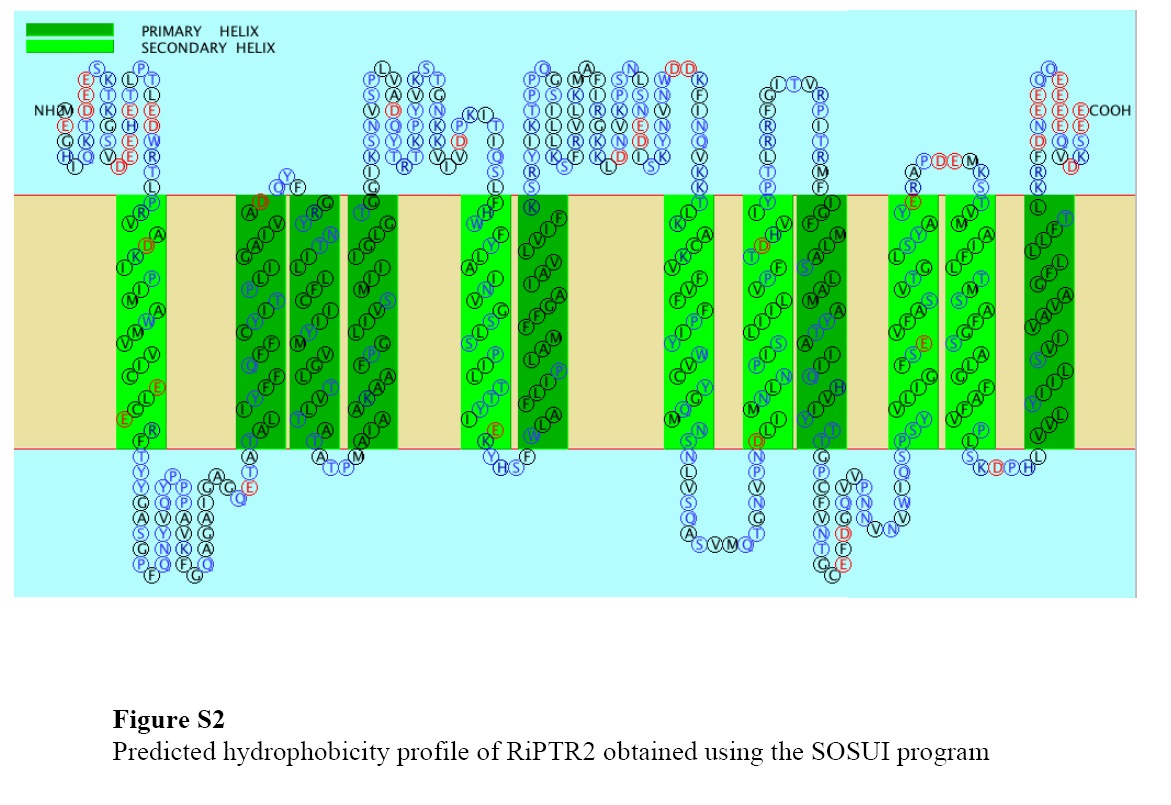

Supplement: Supplementary file 3 [file Image2.JPEG]

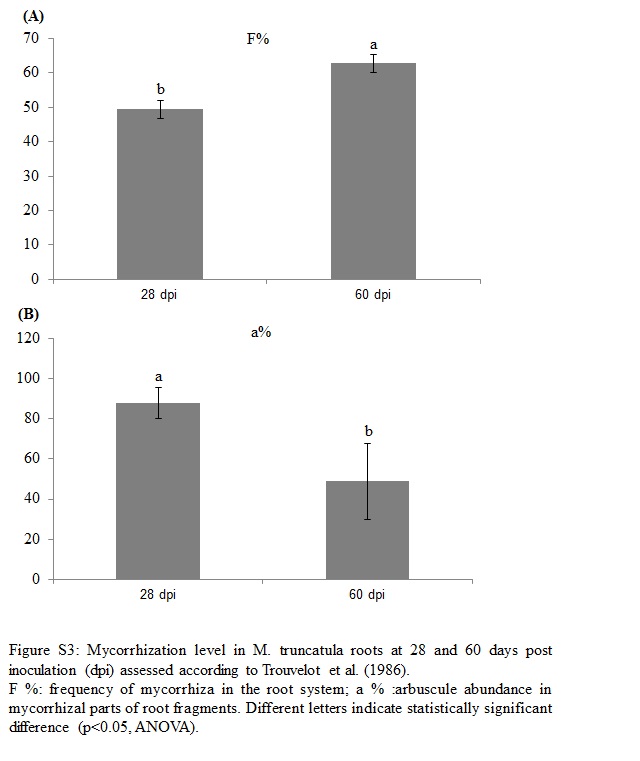

Supplement: Supplementary file 4 [file Image3.JPEG]
